# The Role of the AT-Rich Interaction Domain 1A Gene (*ARID1A*) in Human Carcinogenesis

**DOI:** 10.3390/genes15010005

**Published:** 2023-12-19

**Authors:** Jing Jing Li, Cheok Soon Lee

**Affiliations:** 1 Department of Anatomical Pathology, Liverpool Hospital, Liverpool, NSW 2170, Australia; soon.lee@westernsydney.edu.au; 2 Ingham Institute for Applied Medical Research, Liverpool, NSW 2170, Australia; 3 Discipline of Pathology, School of Medicine, Western Sydney University, Sydney, NSW 2560, Australia; 4 South Western Sydney Clinical School, University of New South Wales, Liverpool, NSW 2170, Australia; 5 Department of Tissue Pathology and Diagnostic Oncology, Royal Prince Alfred Hospital, Camperdown, NSW 2010, Australia

**Keywords:** *ARID1A*, SWI/SNF, BAF, synthetic lethality, tumour suppressor, cancer

## Abstract

The switch/sucrose non-fermentable (SWI/SNF) (SWI/SNF) complex uses energy from ATP hydrolysis to mobilise nucleosomes on chromatin. Components of SWI/SNF are mutated in 20% of all human cancers, of which mutations in AT-rich binding domain protein 1A (*ARID1A*) are the most common. *ARID1A* is mutated in nearly half of ovarian clear cell carcinoma and around one-third of endometrial and ovarian carcinomas of the endometrioid type. This review will examine in detail the molecular functions of ARID1A, including its role in cell cycle control, enhancer regulation, and the prevention of telomerase activity. ARID1A has key roles in the maintenance of genomic integrity, including DNA double-stranded break repair, DNA decatenation, integrity of the cohesin complex, and reduction in replication stress, and is also involved in mismatch repair. The role of ARID1A loss in the pathogenesis of some of the most common human cancers is discussed, with a particular emphasis on gynaecological cancers. Finally, several promising synthetic lethal strategies, which exploit the specific vulnerabilities of ARID1A-deficient cancer cells, are briefly mentioned.

## 1. Introduction

**Figure 1:**
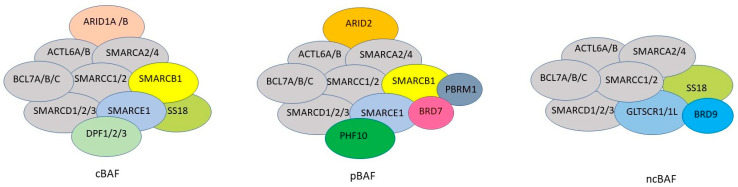
Subunits of the three mammalian SWI/SNF complexes: canonical BAF (cBAF), polybromo-associated BAF (pBAF), and non-canonical BAF (ncBAF). Common subunits are in grey. cBAF uses ARID1A or ARID1B to bind to DNA, while pBAF uses ARID2 to bind to DNA. cBAF and pBAF share SMARCB1 and SMARCE1 subunits. SS18 is only present in cBAF and ncBAF. The bromodomain-containing subunits, PBRM1 and BRD7, are unique to pBAF. ncBAF does not have an ARID subunit but uniquely contains the GLTSCR1/1L and BRD9 subunits. cBAF contains the double plant homeodomain fingers 1/2/3 (DPF1/2/3) subunit, while pBAF contains the plant homeodomain finger 10 (PHF10) subunit.

The switch/sucrose non-fermentable (SWI/SNF) complexes are evolutionally conserved complexes that use energy from ATP hydrolysis to slide or eject nucleosomes at promoters and enhancers. As such, they regulate chromatin accessibility and gene transcription and play a central role in cellular differentiation and lineage specificity [1,2,3,4]. Mammalian cells have three SWI/SNF complexes: canonical Brahma-associated protein (cBAF), polybromo-associated BAF (pBAF), and the recently discovered non-canonical BAF (ncBAF) [1,2]. All three complexes contain the three core subunits SMARCC1, SMARCC2, and SMARCB1, and the catalytic ATPase subunit SMARCA2 or SMARCA4. cBAF complexes contain AT-rich binding domain (ARID) protein, either ARID1A or ARID1B, for binding to DNA; pBAF complexes contain ARID2 instead of ARID1A/1B. There are also subunits that are unique to the three complexes: ss18 is only present in cBAF and ncBAF; the two bromodomain-containing subunits, polybromo 1 (PBRM1) and bromodomain-containing 7 (BRD7), are unique to PBAF; and ncBAF complexes contain GLTSCR1 (glioma tumour suppressor candidate region 1) or GLTSCR1L (GLTSCR1-like) and the BRD9 (bromodomain-containing 7) subunit, instead of ARID1 or ARID2 (Figure 1).

Components of SWI/SNF complexes are mutated in nearly 25% of all cancers [3]. Biallelic loss of *SMARCB1* underlies the key genetic abnormality in atypical rhabdoid/teratoid tumour (AT/RT) and malignant rhabdoid tumour, both aggressive cancers of childhood [5], as well as epithelioid sarcoma [6]. Loss of *SMARCA4* is detected in almost all small cell carcinoma of hypercalcaemic type, an aggressive ovarian neoplasm in young women [7]. Loss of *SMARCA4* is also seen in subsets of lung, oesophageal, and pancreatic carcinomas with rhabdoid appearance and aggressive biology [8]. *SS18-SSX* translocation characterises synovial sarcoma [6].

Of the SWI/SNF components, mutations in *ARID1A* are the most common in human cancers. ARID1A is mutated in 9% of all cancers based on a survey of 24 whole exome studies across 18 different cancer types, followed by PBRM1 (4%) and SMARCA4 (3%) [9]. Most mutations in *Arid1a* are frameshift or non-sense mutations that result in the loss of protein, manifested by negative immunohistochemical staining, in keeping with its function as a tumour suppressor [10]. This review will examine the molecular mechanisms of ARID1A as a tumour suppressor, the role of ARID1A in human cancers, and some of the synthetically lethal strategies that are in the process of development to target tumours of *ARID1A* mutations.

## 2. Cellular and Molecular Functions of ARID1A

### 2.1. ARID1A and Cell Cycle Control (Figure 2)

ARID1A is essential for normal cell cycle arrest in the MC3T3-E1 preosteoblast cell line, which goes through a tightly regulated process of differentiation-associated cell cycle arrest [11]. ARID1A does so via the induction of p21 and the repression of E2F target genes, such as cyclin-dependent kinase 1 (CDK1), cyclin A, and cyclin B [11]. In preosteoblast cell lines, the effect on E2F target genes is by direct suppression at the promoter level, while the induction of p21 is indirectly mediated by the suppression of expression of c-*myc*, which normally acts to inhibit p21 expression [12]. Similarly, ARID1A induces p21 expression in multiple other cell lines, including an ovarian surface epithelial cell line (OSE4), a colorectal cancer cell line (HCT116), a mammary epithelial cell line (MCF10A), and an endometrial cancer cell line (HEC-1A) [13] In these cell lines, ARID1A interreacts with p53 to directly suppress p21 expression at the promoter level [13]. Thus, when ARID1A is deficient, c-*myc* is not suppressed, p21 is not induced, and there is no suppression of the E2F responsive genes such as cyclins and cyclin-dependent kinases, leading to cell cycle progression and cell proliferation.

**Figure 2:**
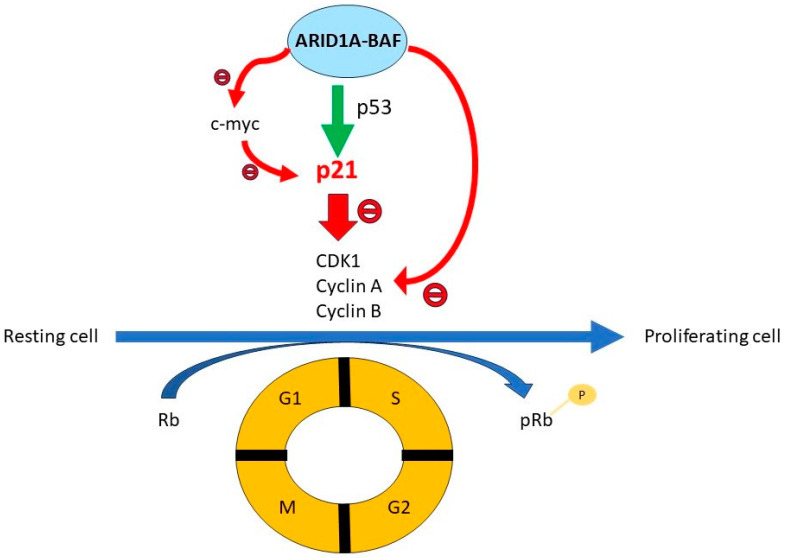
ARID1A and cell cycle control. In normal cells, the cell cycle is a tightly regulated process. The cyclins and cyclin-dependent kinases (CDK) phosphorylate the retinoblastoma protein (Rb), inactivating it, thereby releasing the brake on the G1-S transition. The p21 protein is a CDK inhibitor that can inhibit cell cycle progression. The ARID1A-BAF complex regulates the cell cycle by inducing p21. It does so via a direct effect on p21, where it interacts with p53 on the p21 promoter to induce p21 expression, and an indirect effect via the suppression of c-*myc* transcription. In addition, the ARID1A-BAF complex also suppresses the expression of genes involved in cell cycle progression (the E2F responsive genes), including CDK1, cyclin A, and cyclin B.

### 2.2. ARID1A and Regulation of Promoters and Enhancers (Figure 3)

SWI/SNF complexes are known to be able to mobilise nucleosomes on chromatin via ATP hydrolysis, increase chromatin accessibility, and facilitate gene transcription at promoters [14].

Recent evidence shows that they are also critical for enhancer-regulated gene expression. Certain histone modifications are associated with the activity of promoters and enhancers. The H3K4me3 histone mark is associated with active promoters, H3K4me1 is associated with active and poised enhancers, and H3K27Ac with active promoters and enhancers [15,16]. Multiple studies have demonstrated that, in mouse embryonic fibroblast system and human colorectal cancer cell lines, ARID1A mediates chromatin accessibility, SWI/SNF binding, and deposition of H3K27Ac active histone marks at enhancers [16,17]. The SWI/SNF complex catalysed the histone acetylation via direct interaction with the H3K27 acetyltransferase p300 protein [15]. Loss of ARID1A led to reduced SWI/SNF binding, reduced H3K27Ac histone marks, and loss of gene expression across thousands of enhancers. The binding of AP1 transcription factors was particularly affected by the loss of the H3K27Ac histone mark. In contrast, promoters were relatively unaffected. Similar roles in enhancer regulation have been described for other components of the SWI/SNF complex [18,19].

Thus, the ARID1A SWI/SNF complex modulates the expression of thousands of genes by facilitating chromatin accessibility and controlling the activity of promoters and enhancers. When ARID1A is lost, the expression of thousands of genes is affected, including many tumour suppressor genes and genes of cellular differentiation, stemness, epithelial–mesenchymal transition, and lineage specificity, with massive downstream ripple effects, causing reprogramming of cell identity and oncogenesis.

**Figure 3:**
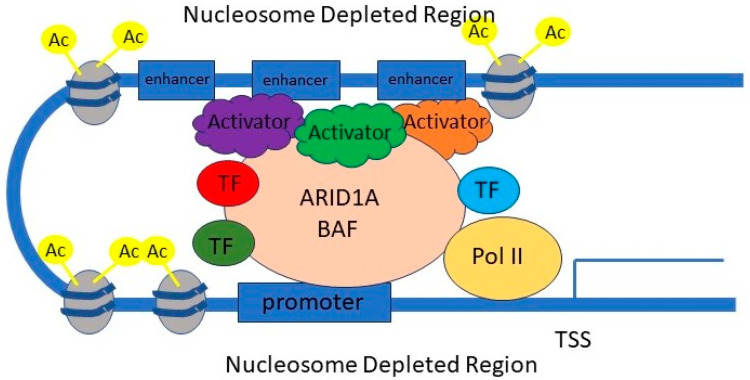
The role of ARID1A in promoter and enhancer regulation. The ARID1A-containing SWI/SNF complex mobilises nucleosomes and creates nucleosome-depleted regions where transcription factors (TF) and activators can bind, enabling gene transcription. It also facilitates acetylation of K27 residues on histone 3 (H3K27), which is associated with promoter and enhancer activation. TSS: transcription start site. TF: transcription factor. Pol II: RNS polymerase II.

### 2.3. ARID1A and Maintenance of Genomic Integrity

#### 2.3.1. ARID1A and DNA Damage Repair

Different types of DNA damage are repaired via a variety of mechanisms, including base excision repair, nucleotide excision repair, mismatch repair (MMR), single-stranded break repair (SSBR), and double-stranded break repair (DSBR) [20].

Double-stranded breaks are the most serious and potentially lethal form of DNA damage. They are repaired by two main pathways, nonhomologous end joining (NHEJ) and homologous recombination (HR). NHEJ is primarily active during the G1 phase of the cell cycle. It ligates the broken DNA ends but is error-prone. HR is much more accurate compared to NHEJ and is primarily active during the S and G2/M phases of the cell cycle. It involves resecting the ends of the damaged DNA and then using a sister chromatid or homologous chromosome as a template to repair the damaged DNA [21].

##### Homologous Recombination (HR) and the Role of ARID1A (Figure 4)

**Figure 4:**
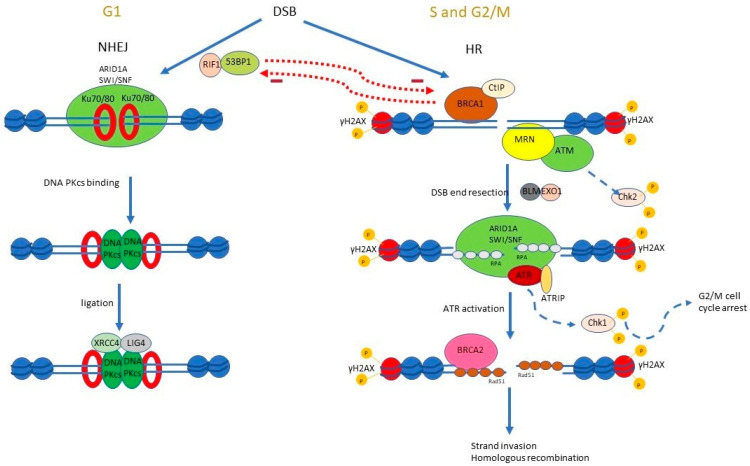
ARID1A and DNA DSB repair. There are two main pathways of DSB repair: NHEJ and HR. In HR, ARID1A binds to DSB by interacting with ATR and facilitates DSB end resection and ATR activation. ARID1A is required for NHEJ, though the mechanism is less clear.

In HR, DSBs are recognised by the MRE11-RAD50-NBS1 (MRN) complex, which activates ataxia-telangiectasia mutated (ATM) serine/threonine kinase. ATM phosphorylates a variety of target proteins, such as checkpoint kinase 2 (Chk2), as well as Ser139 on histone H2AX called γH2AX. γH2AX spreads for distances of up to 1–2 megabases around DSB, and this propagation of γH2AX recruits and stabilises the proteins involved in DNA damage repair. The initial assembly of breast cancer gene 1 (BRCA1) and carboxy-terminal binding protein interacting protein (CtIP) with MRN facilitate limited DSB end resection. In the next step, extensive 5′ DSB end resection is carried out by the Bloom helicase (BLM)-Exonuclease 1 (EXO1) complex, which generates 3′ DNA overhangs that are coated by replication protein As (RPAs). The ssDNA overhangs coated by RPA recruit ataxia-telangiectasia and Rad3-related protein (ATR), which in combination with ATR interacting protein (ATRIP), phosphorylate a wide variety of target proteins such as checkpoint kinase (Chk1). In this next stage of HR, Rad51 displaces RPA on the ssDNA overhangs in a BRCA2-mediated process, followed by strand invasion and accomplishment of HR [22,23] (Figure 4).

ARID1A is recruited to DSB breaks via its interaction with ATR. ARID1A is required for proper chromatin configuration around DSB, and it facilitates DSB end resection to generate RPA-coated ssDNA ends and the subsequent activation of ATR. Without ARID1A, there is impaired activation of ATR, reduced phosphorylation of Chk1, impaired G2/M cell cycle arrest, and defective homologous recombination (Figure 4) [24].

##### Nonhomologous End Joining (NHEJ) and the Role of ARID1A (Figure 4)

In G1, 53BP1 and RIF1 antagonise BRCA1 and suppress the 5′ resection of DSB, thus preventing HR [22]. The Ku heterodimer (Ku70/Ku80) rapidly binds the DSB and activates the catalytic subunit of DNA-protein kinase (DNA-PKcs) to initiate DSB repair. X-ray repair cross-complementing protein 4 (XRCC4)—DNA ligase 4 (LIG4) is subsequently recruited, and this ligates the broken DNA ends (Figure 4).

Deficiency of ARID1A, ARID1B, and several other members of the SWI/SNF complex, reduced the accumulation of proteins involved in NHEJ, including 53BP1, RIF1, Ku70/80, and XRCC4, reduced NHEJ activity at laser-irradiated DSBs, and increased cellular sensitivity to radiation [25,26]. The exact molecular mechanism of involvement of ARID1A in NHEJ, however, has not been elucidated.

##### Mismatch Repair and the Role of ARID1A (Figure 5)

Mismatch repair (MMR) corrects single base pair mismatches and insertion–deletion loops that arise during DNA replication [27,28]. The four main mismatch repair proteins in humans are MSH-2, MSH-6, MLH-1, and PMS-2 [27,28]. MSH-2 is the obligatory partner that forms a complex with MSH-6, called MutSα. MutSα recognises and binds to the mispaired base, exchanges ATP for ADP, and forms a sliding clamp around the DNA mismatch. The MLH-1-PMS-2 heterodimer, called MutLα, is subsequently recruited, and the mispaired base is excised and the correct base synthesised [27,28]. Deficient MMR (dMMR) may be caused by germline mutations in *MSH-2*, *MSH-6*, *MLH-1,* or *PMS-2*, as in Lynch syndrome, or by sporadic hypermethylation of the *MLH-1* promoter. dMMR cancers show microsatellite instability (MSI) and have a hypermutated phenotype. They have increased neoantigen expression, increased tumour infiltrating lymphocytes (TILs), and increased expression of programmed death protein 1 (PD-1) and programmed death-ligand 1 (PD-L1) in TILs and tumour cells, indicating heightened adaptive immune resistance, which could be targeted by immune checkpoint inhibitor therapy [29].

An analysis of TCGA data showed that ARID1A deficiency is associated with higher mutation load across multiple cancer types and is enriched in dMMR/MSI cancers [30]. Subsequent clinical studies have confirmed that ARID1A-deficiency is enriched in MSI cancers of the endometrium [31], stomach [32] and colorectum [33]. Given the high mutation load in MSI cancers, it is possible that the increased frequency of ARID1A deficiency may be due to ‘passenger’ mutations. However, the mutation rate of *ARID1A* is 12- to 61-fold higher than the background mutation rate in MSI gastric cancers [32]. There is also in vitro evidence showing that ARID1A is essential for MMR [30]. Knockdown of ARID1A impaired MMR and increased the mutation load in multiple cancer cell lines. Conversely, restoration of ARID1A rescued MMR and reduced cancer cell mutability. ARID1A does not affect the expression of MSH-2, MSH-6, MLH-1, or PMS-2, but regulates MMR via physical interaction with MSH-2. Given the central role MSH-2 plays in detecting and initiating MMR, this may explain the requirement for ARID1A in MMR and the higher prevalence of ARID1A deficiency in dMMR cancers. Mouse xenografts of ARID1A-deficient ovarian cancers showed greater expression of CD8 and PD-L1, and a superior response to anti-PD-L1 therapy compared to ARID1A-intact tumours [30].

**Figure 5:**
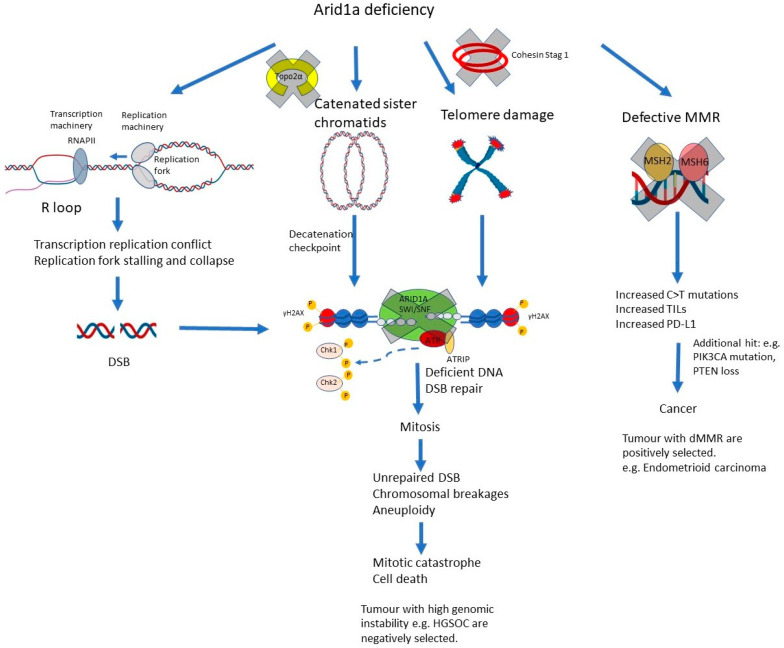
ARID1A and genomic stability. ARID1A loss predisposes the cells to telomere damage due to transcription downregulation of *Stag1*, a component of the cohesin complex. There is reduced recruitment of Topo2α, which leads to DNA catenation and activation of the decatenation checkpoint. There are increased R loops, transcription replication conflict, and replication stress. All these processes lead to increased DSB and chromosomal breakages. In the face of ARID1A deficiency, the DSB repair machinery is non-functional, and the cells go into mitoses carrying these DSB and chromosomal breakages, resulting in mitotic catastrophe and cell death. This may explain why tumours with high genomic stability and copy number aberrations show a low prevalence of ARID1A loss. Aside from this, ARID1A is required for the function of the MSH2-MSH6 heterodimer. ARID1A loss leads to defective MMR, and additional mutations initiate carcinogenesis. This may explain the higher prevalence of ARID1A loss in MMR-deficient cancers. TILs: tumour infiltrating lymphocytes.

#### 2.3.2. ARID1A and DNA Decatenation (Figure 5)

Topoisomerase 2α (Topo2α) modifies the DNA topology in DNA transcription, replication, and cell division. Topoisomerase 2α separates and untangles the two sister chromatids during DNA replication in a process called decatenation. Failure of DNA decatenation activates the decatenation checkpoint, resulting in G2/M arrest, a protective mechanism that allows time for untangling of the sister chromatids before proceeding to mitosis. The decatenation checkpoint is distinct from the DNA damage checkpoint, though components of the latter, including ATR and BRCA, are also involved. When Topo2α is inhibited, cells proceed through mitosis with entwined sister chromatids, leading to anaphase bridges, and chromosomal breakages and aberrations [34,35].

Evidence suggests that SMARCA4 (BRG1) and ARID1A components of the SWI/SNF complex are essential for DNA decatenation by Topo2α and facilitate the binding of Topo2α to chromatin [36,37]. In the absence of functional BRG1 or ARID1A, the cells behave in a manner akin to that of Topo2α inhibition, with failure of decatenation, increased G2/M arrest (reflecting the activation of the decatenation checkpoint), and increased anaphase bridges.

#### 2.3.3. ARID1A and the Cohesin Complex (Figure 5 and Figure 6)

In addition to its role in regulating Topo2α, ARID1A maintains genomic stability through regulation of the cohesin complex, via transcriptional upregulation of *Stag1.*

The cohesin complex is a four-subunit complex that is composed of Smc1, Smc3, Rad21, and either STAG1 or STAG2. The cohesin complex forms a ring-like structure that encircles the two sister chromatids to ensure their cohesion through S and G2, until the sister chromatids become separated in anaphase of mitosis. The cohesin complex is essential for proper sister chromatid alignment and segregation during mitosis [38]. Of the two STAG proteins, Stag1 is responsible for telomere cohesion and Stag2 for centromere cohesion, and both function in chromosome arm cohesion [39].

*ARID1A* knockout in ovarian clear cell carcinoma (OCCC) RMG1 cells and *ARID1A*-mutated OCCC cell lines showed defective telomere cohesion, increased telomere damage, and increased chromosomal defects during mitosis [40]. These effects are mediated through the transcriptional downregulation of *Stag1*. Paradoxically, these cells with defective telomere cohesion tended to undergo apoptosis and are negatively selected for during tumour growth. This may explain the seeming paradox that ARID1A-deficient tumours tended to show fewer gross chromosomal aberrations compared to ARID1A-proficient tumours [40].

**Figure 6:**
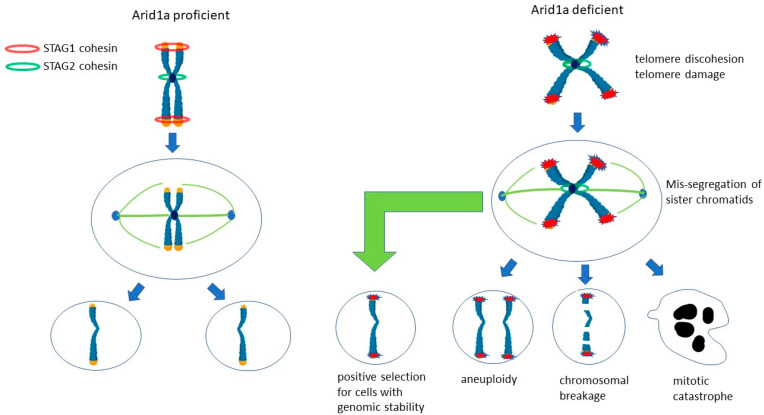
ARID1A and the cohesin complex. The cohesin complex mediates the cohesion of sister chromatids to ensure their proper segregation during mitosis. Stag1 mediates telomere cohesion, whereas Stag2 mediates centromere cohesion. Arid1a loss leads to downregulation of *Stag1*, resulting in telomere discohesion, mis-segregation of sister chromatids at mitosis, and chromosomal aberrations. The cells with chromosomal aberrations are negatively selected, explaining the seeming paradox that Arid1a-deficient cells tend to have greater genomic stability.

#### 2.3.4. ARID1A and Replication Stress (Figure 5)

Replication stress is any stimulus or obstacle that can interfere with DNA replication and cause replication fork stalling and collapse. Replication stress can be caused by ssDNA breaks, DNA lesions, persistent oncogene (e.g., *c-myc*) activation, unusual DNA structures, and heterochromatin, amongst others. Replication stress, and the resultant replication fork stalling/collapse, can result in DSBs, which, if unrepaired, are potentially mutagenic, and replication stress has been linked with genomic instability and tumorigenesis [41,42,43].

In addition to the factors mentioned above, collisions between the replication and transcription machinery (transcription–replication conflict) are also a source of replication stress and can lead to replication fork stalling and collapse. In particular, during transcription, the nascent RNA molecule may hybridise with its complementary DNA strand forming an RNA-DNA hybrid and displacing the non-template strand of unpaired ssDNA (Figure 5). This structure is known as an R-loop and can lead to transcription–replication conflict and stalling of the replication machinery. Normally, a system of helicases (which untangle the DNA-RNA hybrids), topoisomerases (which reduce the negative supercoiling of the DNA molecule), and RNase H (which removes the RNA-DNA hybrids), work to reduce R-loop formation. When these functions are perturbed, however, excessive R-loops form, leading to replication stress and DSBs [42].

There is emerging evidence that ARID1A may be implicated in R-loop regulation. *ARID1A* knocked-out cells show increased R-loop formation, transcription replication conflict, markers of replication stress, and increased DNA damage. The mechanisms via which loss of ARID1A generates R-loops are unknown and one possibility is via reduced localisation of topoisomerase 2ɑ to specific R-loop sites [44].

#### 2.3.5. Summary of the Role of ARID1A in Genomic Integrity

ARID1A is a key component of DSB repair, especially in the HR pathway. It maintains genomic integrity by facilitating DNA decatenation, maintaining the integrity of the cohesin complex, and preventing replication stress. Without ARID1A, DSBs resulting from these aberrant processes are unrepaired, leading to mitotic catastrophe and cell death. This may explain the paradox of why tumours with high genomic instability, such as high-grade serous ovarian carcinoma (HGSOC), have a low prevalence of ARID1A (Figure 5), as loss of the ARID1A-BAF complex would render these tumours non-viable.

In contrast, ARID1A facilitates mismatch repair (MMR) via a functional interaction with MSH2, and this may explain why MMR-deficient cancers, such as subsets of endometrioid uterine carcinoma, and gastric and colorectal carcinoma, show greater frequency of ARID1A loss (Figure 5). This has therapeutic implications, given the known association of MMR-deficient cancers with greater host immune activation and enhanced checkpoint inhibitor sensitivity [29].

### 2.4. ARID1A and Prevention of Telomere Lengthening

Cancer cells have mechanisms that maintain telomere lengths, thereby preventing telomere shortening, replicative senescence, and cell death. They do this by either activating the telomerase, encoded by the telomerase reverse transcriptase (*TERT*) gene, which lengthens telomeres, or by the alternative lengthening of telomeres (ALT) pathway, which maintains telomere length by homologous recombination. The most common *TERT* mutations are the *C228T* and *C250T* activating mutations in the *TERT* promoter. These mutations are highly prevalent in human cancers, including urothelial cancer, melanomas, and glioblastomas [45]. *TERT* promoter mutations, however, tend to be mutually exclusive with loss of ARID1A protein expression in ovarian clear cell carcinoma (OCCC) [46], which suggested that tumours with ARID1A loss may have other mechanisms for the maintenance of telomere lengths. Indeed, ARID1A, in combination with the Sin3A histone deacetylase complex, binds to the *TERT* promoter and represses *TERT* transcription. ARID1A knockdown or knockout in cell lines led to increased *TERT* expression, telomerase activity, and increased telomere length [47].

## 3. ARID1A in Human Carcinogenesis

The significance of *Arid1a* mutations in gynaecological cancers was discovered in 2010 when mutations in *Arid1a* were found in almost half of ovarian clear cell and endometrioid carcinomas [48,49]. The role of ARID1A in some of the most common human cancers is discussed below.

### 3.1. ARID1A in Gynaecological Cancers

**Figure 7:**
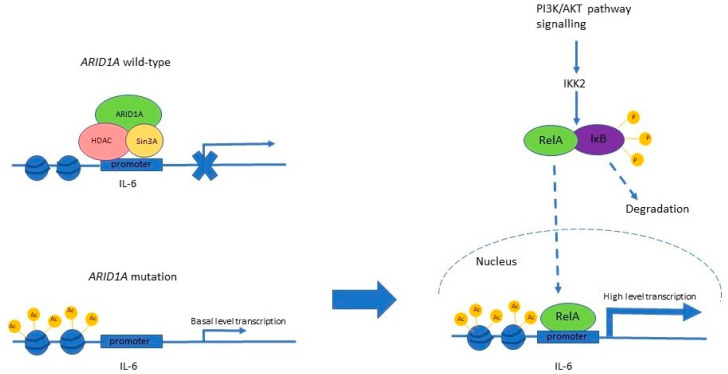
ARID1A deficiency and *PIK3CA* mutation interact to promote tumorigenesis of OCCC via upregulation of *IL-6*. ARID1A recruits the histone deacetylase (HDAC) and Sin3A repressive complex to the *IL-6* promoter and inhibits its expression. When ARID1a is lost, there is de-repression of IL-6 expression, leading to low-level basal transcription. Activation of the PI3K/AKT pathway, by *PIK3CA* mutation, leads to downstream activation of catalytic IKK2 subunit in the IKK (IкB kinase) complex, which phosphorylates IкB, leading to its proteasomal degradation. This releases RelA, an NF-кB, to translocate to the nucleus, where it binds the now de-repressed IL-6 promoter, leading to high-level transcription of the gene.

Clear cell carcinoma of the ovary tends to occur in younger women and is associated with endometriosis [50]. *ARID1A* mutations are found in half of ovarian clear cell carcinoma (OCCC) [48,49]. In OCCC, *ARID1A* mutation is often found in contiguous endometriosis and is an early event in neoplastic transformation [49,51,52]. In vitro, expression of ARID1A in ARID1A-deficient ovarian cancer cell lines reduced cellular proliferation, the percentage of cells in the S phase, and the growth of tumour xenografts [13]. Knockdown of *ARID1A* in ovarian surface epithelial cell lines increased cell proliferation, percentage of cells in the S phase, and tumorigenicity [13]. *ARID1A* and *PIK3CA* mutations are frequently found together in OCCC [51,53], and they are synergistic in tumour formation. In an animal model, mice with both *ARID1A* homozygous deletion and *H104R* activating mutation of *PIK3CA* in ovarian surface epithelium rapidly developed ovarian tumours with haemorrhagic ascites and peritoneal metastases, whereas those with *ARID1A* deletion or *PIK3CA* mutations alone did not [53]. The resulting ovarian tumours resembled human OCCC. The proinflammatory cytokine interleukin 6 (IL-6) was overexpressed in OCCC with concurrent *ARID1A* deletion and *PIK3CA* mutation (Figure 7), and the cytokine promoted tumour cell growth and survival [53].

*ARID1A* mutation or loss is found in 25% to 50% of endometrioid endometrial adenocarcinoma [10,54]. The prevalence of ARID1A loss increases with the grade of the tumour, suggesting a role in tumour progression [54]. The finding of ARID1A loss in complex atypical hyperplasia of endometrial curettage is highly predictive of endometrioid adenocarcinoma at surgery [55].

The Cancer Genome Atlas (TCGA) Research Network classifies endometrial carcinoma into four molecular groups: DNA polymerase epsilon, catalytic subunit (*POLE*) mutant or ultramutated (7%), microsatellite unstable (MSI) or hypermutated (28%), no specific molecular profile (NSMP) (also known as p53 wild-type, copy-number-low, or endometrioid) (39%), and p53 mutant (also known as copy-number-high, or serous like) (26%) [56]. These groups have distinct clinical, pathological, and prognostic characteristics [57,58,59]. Loss of ARID1A is most seen in the MSI group (63.7%), followed by NSMP (40.8%), and is least common in the p53 mutant group (27%) [58]. In the first two groups, *ARID1A* mutations commonly occur with mutations of genes in the PI3K/PTEN axis, including mutations in *PIK3A* and *PTEN* [56]. *ARID1A* is likely to be pathogenic rather than a ‘passenger’ mutation in these MSI endometrial cancers, as ARID1A loss was found in only 14% of Lynch syndrome MSI endometrial carcinomas compared to 75% of sporadic MSI endometrial carcinomas in one study [60]. There is also abundant in vitro and animal evidence of the pathogenic role of *ARID1A* loss and its synergism with *PIK3CA* mutations and *PTEN* loss in vitro and in animal models. Expression of *ARID1A* in *ARID1A*-deficient uterine endometrioid carcinoma cell lines reduced cellular proliferation, the percentage of cells in the S phase, and the growth of tumour xenografts [13]. Conversely, *ARID1A* knocked-out human endometrial cells exhibited reduced transformation growth factor-β (TGF-β) pathway signalling, reduced response to inhibitory effects of TGF-β on cell motility, and increased cellular invasiveness. This effect is mostly likely mediated through reduced chromatin accessibility, reduced binding of the SWI/SNF complex, and consequent downregulation of TGF-β pathway genes in *ARID1A* knocked-out cells [61]. Mice with a double knockout of *ARID1A* and *PTEN* in the uterine epithelium developed a rapidly progressive invasive endometrial carcinoma, while *ARID1A* deletion alone could not initiate neoplastic transformation, and *PTEN*-deleted mice developed predominantly intraepithelial epithelial neoplasia which only slowly progressed to early endometrioid carcinoma [61]. Similarly, homozygous or heterozygous ARID1A loss in the mouse endometrial epithelium, when combined with *PIK3CA*^H1047R^ mutation, led to the development of endometrial carcinoma [62]. Both the mouse model and the in vitro model of *ARID1A* knocked-out *PIK3CA* H1047R mutated human endometrial cell line showed increased accessibility at promoters and upregulation of genes involved in epithelial–mesenchymal transition (EMT) and enhanced invasive properties of the endometrial cells [62].

Downregulation of progesterone receptor (PGR) in endometrial carcinoma leads to progestin resistance and is a poor prognostic factor. Recent evidence suggests that ARID1A may have a role in progesterone insensitivity, as loss of ARID1a led to reduced expression of PGR in human endometrial cell lines, mouse models, and clinical tumour tissues [63,64]. When ARID1A is lost, there is reduced PGR expression, due to both reduced H3K27Ac acetylation and SWI/SNF binding at the PGR enhancer [64], and unopposed polycomb repressive complex 2 (PRC2) activity [63].

Endometrioid ovarian adenocarcinoma is commonly associated with endometriosis, which may occur with synchronous endometrial carcinoma, has a more favourable prognosis compared to high-grade serous carcinoma, and is driven by similar mutations as endometrial endometrioid carcinoma, including *PIK3CA*, *PTEN*, *CTNNB1*, and *ARID1A* [65]. Like endometrial carcinoma, endometrioid ovarian carcinoma can be classified into the four TCGA molecular groups, *POLE* mutant (5%), MSI (17%), NSMP (66%), and p53 mutant/serous like (11%), with distinct characteristics and prognostic implications [66,67,68]. Like endometrial carcinoma, ARID1A loss occurs most commonly in the MSI (37.9%) and NSMP (16.5%) groups [66], and has a synergistic effect with mutations of the PI3K/PTEN axis. In an animal model, 59% of mice with *ARID1A* and *PTEN* double knockout of mouse ovarian surface epithelium developed ovarian endometrioid or undifferentiated carcinomas, whereas mice with *ARID1A* knockout alone did not. The mouse ovarian tumours bore the closest resemblance to human endometrioid carcinoma based on gene expression profiling analysis [69].

While *ARID1A* mutations are common in endometrioid adenocarcinoma, co-inactivation of ARID1A and 1B, the mutually exclusive subunits of the BAF complex, is required for the formation of dedifferentiated and undifferentiated endometrial carcinoma, which shows undifferentiated, monotonous, occasionally rhabdoid tumour cells on histology, with loss of expression of PAX8 and ER, and clinically aggressive biological behaviour [70,71]. Indeed, loss of BAF components, including dual loss of ARID1A and 1B, loss of SMARCB1, and loss of SMARCA4, are found in more than half of dedifferentiated and undifferentiated endometrial carcinoma, in a mutually exclusive manner [70,71]. The loss of BAF components is thought to abrogate the BAF complex function and prevent cellular differentiation of the carcinoma cells, causing them to be arrested in a permanently undifferentiated state [70,71].

The prognostic impacts of ARID1A on gynaecological cancers are unclear. While a few studies suggest that loss of ARID1A expression is associated with chemoresistance and reduced progression-free survival in OCCC [72,73], a recent large study of an international consortium did not find any prognostic impact [74]. Similarly, there is no clear association between ARID1A loss and prognosis in endometrial carcinoma [75,76].

### 3.2. ARID1A in Gastric Carcinoma

Loss of ARID1A expression occurs in up to 8–27% of gastric adenocarcinoma [77] but is enriched in microsatellite instability (MSI) (83%) and EBV-infected subtypes (73%) [32,78]. Loss of ARID1A in gastric cancer is correlated with advanced stage, tumour invasion, lymphovascular invasion, lymph node metastases, and reduced survival [77,79,80,81,82,83], especially in poorly differentiated and early-stage cases [83].

Loss of ARID1A promotes gastric cancer growth in vitro [32,78,84,85,86]. ARID1A loss is associated with enhanced activation of the PI3K/AKT pathway in vitro [85,86], as well as activation of mTOR signalling and increased expression of SOX9, a gastrointestinal stem cell marker, in tissue microarrays, cell lines, patient xenograft tumours, and mouse models [84]. *ARID1A* knockdown in a *TP53-/-* human gastric organoid model induced dysplasia and tumorigenesis [87]. Knockdown of *ARID1A* in gastric cancer cell lines led to reduced E-cadherin (*CDH1*) expression, disruption of the cadherin/catenin complex, epithelial–mesenchymal transition, and enhanced invasive properties of the cells [88].

### 3.3. ARID1A in Colorectal Cancer

*ARID1A* is mutated in around 8–9% of colorectal adenocarcinoma [89]. Deletion of *ARID1A* in mouse intestines led to the development of colorectal carcinoma via a non-*APC*-dependent pathway [16]. ARID1A deficiency in cell lines and mouse models showed enhancer-mediated dysregulated gene expression [16]. In clinical samples, ARID1A deficiency in colorectal cancer is associated with a right-sided location, microsatellite instability (MSI), medullary histology, higher tumour mutation burden (TMB), higher PD-L1 expression, tumour-infiltrating immune cells, and a higher percentage of consensus molecular subtype I (CMI-1) of the Colorectal Cancer Subtyping Consortium (CRCSC) classification [33,89,90,91,92,93], suggesting that it may respond favourably to immune checkpoint inhibitor therapy [94]. ARID1A deficiency does not have a significant impact on colorectal cancer prognosis [33].

### 3.4. ARID1A in Breast Carcinoma

*ARID1A* mutations occur in 5% of primary breast cancer but are enriched in endocrine-treatment-resistant tumours and metastases (12%) [95]. *ARID1A* was first discovered as a tumour suppressor in breast cancer when Mamo et al. identified a nonsense mutation in *ARID1A* in the T47D breast cancer cell line [96].

Loss of ARID1A expression is associated with higher-grade tumours and triple-negative status [96] and predicts poorer response to paclitaxel in triple-negative breast cancer [97]. In *Her2*-amplified breast cancer cell lines, ARID1A loss is associated with activation of the PI3K/AKT pathway, increased annexin A1 expression, and trastuzumab resistance [98].

**Figure 8:**
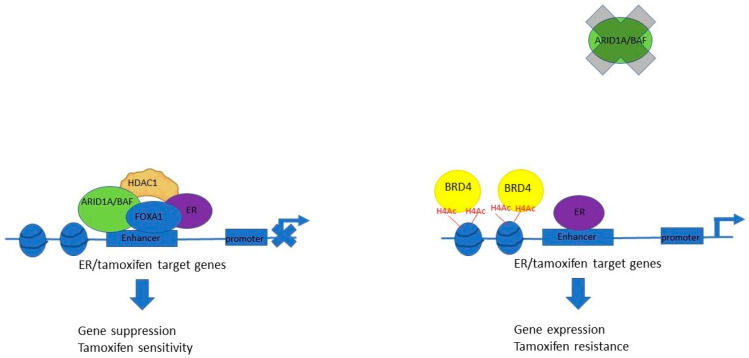
ARID1A mediates tamoxifen sensitivity. ARID1A, as part of the BAF complex, is recruited to the enhancer regulatory elements of ER/tamoxifen target genes via FOXA1. ARID1A interacts with HDAC1, which deacetylates histones. This has a suppressive effect on gene expression and facilitates sensitivity to tamoxifen. When ARID1A is deficient, HDAC1 no longer binds, and acetylation marks are deposited on histone 4. H4Ac marks recruit BRD4, which facilitates gene expression and resistance to tamoxifen. HDAC1: histone deacetylase 1. BRD4: bromodomain-containing protein 4.

*ARID1A* mutations are associated with treatment resistance and inferior survival in patients receiving endocrine therapy [99]. The mechanism of ARID1A in mediating endocrine resistance is starting to be elucidated. Knockdown of *ARID1A* in breast cancer cell lines led to widespread alterations in chromatin accessibility, SWI/SNF binding, and occupancy of the pioneer transcription factor FOXA1-ER complex, as well as transcriptional reprogramming from a luminal to a basal-like gene signature, and resistance to anti-estrogen therapies such as tamoxifen and fulvestrant, both in vitro and in xenograft models [95,99]. FOXA1 is thought to mediate the recruitment of the ARID1A-SWI/SNF complex to estrogen receptor (ER) target genes, where ARID1A suppresses the expression of ER-dependent genes and is essential for tamoxifen efficacy [95], the proposed mechanism of which is illustrated in Figure 8.

### 3.5. ARID1A in Cholangiocarcinoma

*ARID1A* is mutated in 7–36% of cholangiocarcinoma (CC), where it frequently co-occurs with *KRAS* mutations [100]. In a liver-specific mouse model, concurrent *KRAS*^G12D^ mutation and *ARID1A* loss significantly increased the formation of CC and its biliary precursors, compared with either mutation alone. *KRAS*^G12D^ *ARID1A -/-* mouse embryonic fibroblasts had increased proliferative capacity, increased chromatin accessibility at promoters and upregulation of E2F targets, and upstream inhibition of the transformation growth factor β (TGF-β)-SMAD pathway, which leads to unrestrained cholangiocyte proliferation in response to injury [100].

### 3.6. ARID1A in Pancreatic Adenocarcinoma

*ARID1A* is mutated in 10% of intraductal papillary mucinous neoplasms (IPMNs) [101] and 6% of pancreatic adenocarcinoma (PDAC) [102], while *KRAS* mutations are found in more than 95% of pancreatic ductal adenocarcinoma (PDAC) [102]. Concurrent *KRAS^G12D^* mutation and *ARID1A* deletion in mice pancreata led to the formation of cystic lesions resembling IPMN and accelerated development of PDAC [101,103,104]. ARID1A loss induces epithelial–mesenchymal transition in pancreatic ductal adenocarcinoma (PDAC) cell lines, with reduced expression of E-cadherin, increased expression of vimentin, enhanced invasive properties, and upregulation of EMT genes [103,105].

### 3.7. ARID1A in Hepatocellular Carcinoma

*ARID1A* mutations are observed in 10–16.8% of hepatocellular carcinoma (HCC), and 13% of hepatitis-B-virus-associated HCC [106]. ARID1A mutation and loss are associated with adverse prognosis in HCC [106,107,108].

ARID1A loss promotes the migration and invasion of HCC cells in vitro and promotes tumour growth in mouse xenografts and diethylnitrosamine-induced mouse models of HCC [106,108,109,110]. ARID1A has context-dependent roles in mouse models of hepatocellular carcinoma [111]. During tumour initiation, ARID1A is proto-oncogenic, and promotes tumour formation by upregulation of cytochrome P450 proteins and generation of reactive oxygen species. Once the tumours are established, ARID1A acts as a tumour suppressor via downregulation of metastasis-associated genes [112].

ARID1A deficiency may also promote HCC development by altering the tumour microenvironment and vascularity. Hepatocyte-specific ARID1A knockout in mice promoted inflammatory cell infiltration, pro-inflammatory cytokine production, steatohepatitis, and HCC development [109]. ARID1A deficiency promotes elevated expression of angiopoietin 2 and angiogenesis in HCCs [110].

### 3.8. ARID1A in Urothelial Carcinoma

Mutations of SWI/SNF complex genes are seen in 64% of urothelial carcinomas, of which mutations in *ARID1A* are the most frequent, occurring in 13–38% of cases [113,114]. Loss of ARID1A expression was seen in 4 of 14 cases of undifferentiated/rhabdoid urothelial carcinoma in a case series [115]. Loss of ARID1A expression in urothelial carcinoma is associated with higher grade and stage [113,116], but with no prognostic impact [113].

### 3.9. ARID1A in Lung Carcinoma

ARID1A mutations were present in 6–11.3% of non-small-cell lung carcinomas (NSCLC), of which the majority (44–69%) were loss of function mutations [117,118,119,120], while less than 2% showed diffuse loss of expression [118,121]. Diffuse loss of ARID1A expression corresponded to ARID1A loss of function mutations and biallelic inactivation [118]. ARID1A mutations in NSCLC were associated with less frequent *EGFR* mutations, more frequent *TP53* and *KRAS* mutations, and increased tumour mutation burden [118,122]. Loss of ARID1A expression in NSCLC was associated with poorly differentiated histology, smoking status, lymphatic invasion, distant metastasis, higher TNM stage, and predicted reduced overall survival [118,119,123,124,125].

Knockdown of ARID1A in lung adenocarcinoma cell lines promoted cell proliferation, migration, invasion, and enhanced phosphorylation of Akt, and there are enhanced tumour metastases in xenograft models [119]. In a genetically engineered *KRAS^G12D^ TP53-/- ARID1A -/-* mouse model, ARID1A deficiency promoted tumorigenesis of lung adenocarcinoma compared to mice that were wild-type or heterozygous for *ARID1A* deletion [117]. ARID1A recruits histone deacetylase 1 and normally suppresses the expression of genes of the glycolysis pathway. ARID1A deficiency in this mouse model upregulated the expression of glycolysis enzymes and promoted glycolysis, which is tumorigenic [117].

## 4. Synthetic Lethal Strategies

Synthetic lethality is defined as a genetic interaction, where the co-occurrence (or ‘synthesis’) of two genetic events results in organism or cell death [126]. That is, a cell with only mutated/lost gene A or gene B may be able to stay alive, but the loss/mutations of both genes A and B renders the cell non-viable. The principles of synthetic lethality could be used to create novel cancer therapies. Traditional targeted therapies have focused on ‘oncogene addiction’, where cancer cells have come to rely on a mutated, constitutively activated oncogene, usually a tyrosine kinase receptor, and inhibition of this oncogene can suppress tumour cell growth. Mutations in tumour suppressor genes, on the other hand, are traditionally non-targetable, as it is nearly impossible to restore the function of the tumour suppressor. They may, however, be targeted in a synthetic lethal manner, whereby inactivation of another gene product or cellular pathway renders these tumour cells non-viable. Several synthetic lethal strategies are being developed for ARID1A deficient cancers, including PAPR inhibitors [24], EZH2 inhibitors [127], BET inhibitors [128], ATR inhibitors [37], and inhibitors of HDAC2 [129] and 6 [130]. Most of these are in the experimental and preclinical stages, although several clinical trials are underway. A selected few are discussed below.

### 4.1. PARP Inhibitors

The involvement of ARID1A in DSB repair may be exploited therapeutically. Poly(ADP-ribose) polymerases (PARPs), specifically PARP1 and PARP2, are essential for ssDNA break repair. ssDNA breaks are sensed by PARP1/2, which synthesises polyADP-ribose chains from NADH and attaches them to target proteins, including PARP itself, initiating the cascade of events leading to ssDNA break repair. In the presence of PARP inhibitors, ssDNA breaks are not repaired, leading to replication fork stalling/collapse, and generation of DSBs. Normal cells can repair DSBs by HR; however, cells deficient in HR, such as *BRCA 1* or *2* mutant cells, are unable to repair the DSB and die. PARP inhibitors are thus synthetically lethal in tumour cells with *BRCA 1/2* mutations [131].

Given the role ARID1A plays in DSB repair, both HR and NHEJ, one may expect that PAPR inhibitors might be synthetically lethal in ARID1A deficient cells. Indeed, ARID1A deficiency sensitises tumour cells to PARP inhibitors in breast cancer and colorectal cancer cells in vitro and in vivo, and this sensitivity is dependent on the interaction of ARID1A with ATR [24]. Similarly, PARP inhibitors show synergistic cytotoxicity with ionising radiation in ARID1A-deficient colorectal and OCCC cells in vitro and in vivo [26]. Currently, clinical trials for ovarian cancer using ARID1A as a biomarker are underway [132].

### 4.2. EZH2 Inhibitors (Figure 9)

Enhancer of zeste homolog 2 (EZH2) is the enzymatic subunit of polycomb repressive complex 2 (PRC2), which also includes EED, SUZ12, and RbAP46/48. PRC2 catalyses the trimethylation of lysine at position 27 of histone 3 (H3K27me3) at gene promoters, which leads to gene silencing. EZH2 overexpression has been found in a variety of cancers including breast, prostate, endometrial, and melanomas, and is associated with increased tumour aggressiveness. In addition, *EZH2* gain of function mutations are found in 22% of diffuse large B cell lymphoma and 7–12% of follicular lymphoma [133]. EZH2 inhibitors have led to tumour suppression in vitro and in vivo, and there are numerous clinical trials in progress [134].

There is an evolutionally conserved antagonistic relationship between SWI/SNF complexes and PRC2. Loss of SWI/SNF complexes in cancer cells leads to unopposed PRC2 activity, which silences the expression of tumour suppressor genes, driving oncogenesis. This antagonistic relationship between PRC and SWI/SNF is well illustrated for the SMARCB1 deficient malignant rhabdoid tumour [135,136], and EZH2 inhibitors led to regression of malignant rhabdoid tumours in vitro and in vivo [137]. EZH2 inhibition led to impaired proliferation of several human cancer cell lines with mutant SWI/SNF genes including *BRG1 (SMARCA4)*, *PBRM*, and *ARID1A* [138]. Clinical trials of EZH2 inhibitors in malignant rhabdoid tumours, small cell carcinoma of hypercalcaemic type, SMARCA4 deficient thoracic sarcoma, and epithelioid sarcoma are ongoing [139,140].

**Figure 9:**
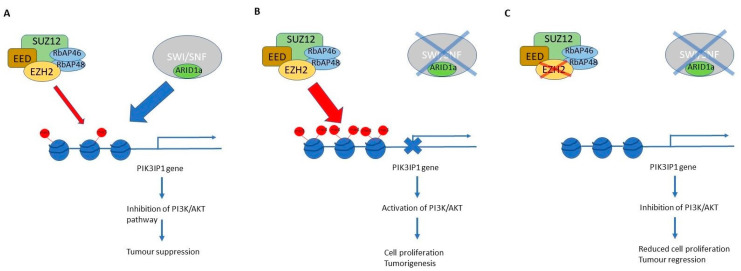
EZH2 inhibitors are synthetically lethal in ARID1A deficient ovarian clear cell carcinoma. (**A**) In the presence of Arid1A and functional SWI/SNF complex, Arid1A overcomes the inhibitory effects of EZH2 and activates the transcription of the *PIK3IP1* gene, which negatively regulates the PI3K/AKT pathway. (**B**) When Arid1a is deficient, EZH2 is unopposed, which trimethylates histone H3K27 and silences the *PIK3IP1* gene. The result is activation of the PI3K/AKT pathway and oncogenesis. (**C**) EZH2 inhibitors relieve the inhibitory activity of PRC2 and remove the H3K27 trimethyl marks, leading to the expression of *PIK3IP1*, which results in tumour regression.

Bitler et al. [127,141] showed that GSK126, a small molecule enzymatic inhibitor of EZH2, was synthetic lethal in *ARID1A*-mutated OCCC in vitro and in vivo. Most of this synthetic lethality is mediated by PIK3IP1, which negatively regulates PI3K/AKT signalling, as follows: In the presence of functional ARID1A SWI/SNF complex, the *PIK3IP1* gene is upregulated and its expression inhibits PI3K/AKT signalling, suppressing cell growth. When ARID1A is mutated, the up-regulatory action of ARID1A is lost, and EZH2 can trimethylate H3K27 and cause suppression of *PIK3P1*. Inhibiting EZH2 removes the trimethylation marks and relieves the suppression of *PIK3IP1*, which is then able to inhibit the PI3K/AKT pathway, leading to tumour suppression (Figure 9). Trials of EZH2 inhibitors in gynaecological cancers, using *ARID1A* as a biomarker, are ongoing [132].

### 4.3. BET Inhibitors

Bromodomain and extraterminal domain (BET) proteins, including bromodomain-containing protein 2 (BRD2), BRD3, and BRD4, bind to acetylated lysine residues on histones near promoters and enhancers, thereby recruiting the mediator complex and the positive transcription elongation factor b (pTEFb), which in turn phosphorylates serine 2 of RNA POL II, leading to enhanced gene transcription [142,143]. BET proteins, such as BRD4, enhance cell proliferation by activation of protooncogenes such as c*-myc* [142,143]. *BRD4-NUT* fusion, and less frequently *BRD3-NUT* fusion, characterise the genetic abnormality seen in NUT carcinoma, an aggressive carcinoma occurring in the midline of the head and neck region [144]. BET inhibitors have shown promising anti-tumour effects in preclinical models of NUT carcinoma and several haematological and visceral malignancies [142,143].

Silencing of *BRD2* is synthetically lethal in several ARID1A mutant OCCC cell lines, and BET inhibitors inhibited the growth in OCCC cells in vitro and in vivo [128]. The effect is partially mediated by BRD2 inhibition by BET inhibitors, which in turn causes the silencing of *ARID1B*, an effect that is synthetically lethal in ARID1A mutant cells [145].

### 4.4. ATR Inhibitors

ATR inhibitors are synthetically lethal in ARID1A-deficient human colorectal cancer and OCCC cell lines and xenograft models [37]. The role of ARID1A in facilitating Topo2α binding and DNA decatenation may explain the synthetic lethality of ATR inhibitors in ARID1A deficient cells, as follows: In ARID1A deficient cells, cells have impaired Topo2α mediated DNA decatenation, and rely heavily on the decatenation checkpoint for extra time to resolve the tangled sister chromatids before entering mitosis. As mentioned above, ATR is essential for the decatenation checkpoint. In the presence of ATR inhibitors, cells cannot use the decatenation checkpoint and proceed to mitosis with tangled chromosomes, resulting in anaphase bridges, chromosomal breakages, and ultimately cell death.

## 5. Conclusions

ARID1A is the DNA binding subunit of the SWI/SNF complex, which uses ATP hydrolysis to mobilise nucleosomes. Through the regulation of chromatin accessibility and histone acetylation at enhancers and promoters, ARID1A regulates the transcription of thousands of genes across the genome. It has a ubiquitous role in cell biology and is involved in diverse processes including DNA damage repair, maintenance of genomic integrity, cell cycle regulation, epithelial–mesenchymal transition, and steroid receptor response. It is the most commonly mutated subunit of the SWI/SNF complex in human cancers and is a bona fide tumour suppressor gene, being particularly important in gynaecological cancers. ARID1A status could be assessed by multiplex next-generation sequencing and/or immunohistochemistry in routine clinical specimens. The high prevalence of ARID1A mutations in MSI cancers suggests that it has the potential to be a biomarker predicting sensitivity to immune checkpoint inhibition, along with tumour mutational burden, and TIL and PD-L1 expression, but this will need validation in large clinical trials. In the next decade, we hope that the targeting of *ARID1A* mutation by synthetic lethality will extend beyond the realms of academic interest and laboratory studies and show demonstrable benefit in clinical trials, and that clinicians at the bedside will be able to use ARID1A as a biomarker to triage patients that may potentially benefit from these novel therapies, offering a chance of improving survival in cancers with otherwise often dismal outlooks.
